# Influence of Catechol-O-Methyltransferase (COMT) Val158Met Polymorphism in Conditioned Pain Modulation in Women with Migraine

**DOI:** 10.3390/ijms27115107

**Published:** 2026-06-04

**Authors:** Margarita Cigarán-Méndez, Ana I. de-la-Llave-Rincón, Juan C. Pacho-Hernández, Angela Tejera-Alonso, Cristina Gómez-Calero, César Fernández-de-las-Peñas, Lars Arendt-Nielsen, Silvia Ambite-Quesada

**Affiliations:** 1 Department of Psychology, Universidad Rey Juan Carlos, 28922 Alcorcón, Spain; margarita.cigaran@urjc.es (M.C.-M.);; 2 Department of Physical Therapy, Occupational Therapy, Physical Medicine and Rehabilitation, Universidad Rey Juan Carlos, 28922 Alcorcón, Spain; anaisabel.delallave@urjc.es (A.I.d.-l.-L.-R.); cristina.gomez@urjc.es (C.G.-C.); silvia.ambite.quesada@urjc.es (S.A.-Q.); 3 Department of Research and Psychology in Education, Universidad Complutense de Madrid, 28040 Madrid, Spain; jpacho@ucm.es; 4 Center for Neuroplasticity and Pain (CNAP), Sensory-Motor Interaction (SMI) Center, Department of Health Science and Technology, Faculty of Medicine, Aalborg University, DK-9220 Aalborg, Denmark; lan@hst.aau.dk; 5 Department of Gastroenterology & Hepatology, Mech-Sense, Clinical Institute, Aalborg University Hospital, DK-9000 Aalborg, Denmark; 6 Steno Diabetes Center North Denmark, Clinical Institute, Aalborg University Hospital, DK-9000 Aalborg, Denmark

**Keywords:** migraine, conditioned pain modulation, Val158Met, Met allele, genetics

## Abstract

The role of the catechol-O-methyltransferase (COMT) Val158Met rs4680 polymorphism in altered pain processing in headaches is controversial. The aim of this study was to investigate the influence of the Val158Met rs4680 polymorphism in conditioned pain modulation (CPM) in women with migraine. A case-control study including 70 women with chronic migraine, 70 with episodic migraine and 70 pain-free women was conducted. Pressure pain thresholds (PPTs) at the temporalis muscle, lateral epicondyle, and tibialis anterior were bilaterally assessed. Heat (HPT) and cold (CPT) pain thresholds at the frontalis muscle were also assessed. Subsequently, CPM was evaluated immediately after a one-minute cold-pressor test paradigm on changes obtained in PPTs, HPT and CPT. Thus, after amplifying Val158Met polymorphism by polymerase chain reaction, genotype frequencies (Val/Val, Val/Met, or Met/Met) and allele distributions were identified. The distribution of Val158Met genotypes (*p* = 0.097) was not significantly different among women with episodic migraine, chronic migraine and pain-free controls. The results revealed significant group*time*Val 158Met interactions for PPTs at the temporalis (Wilk’s λ = 0.917, F [4, 193] = 4.377, *p* = 0.002, n^2^p = 0.083, 1 − β = 0.930) and lateral epicondyle (Wilk’s λ = 0.892, F [4, 193] = 5.870, *p* < 0.001, n^2^p = 0.108, 1 − β = 0.982), as well as CPT (Wilk’s λ = 0.872, F [4, 193] = 7.111, *p* < 0.001, n^2^p = 0.128, 1 − β = 0.995) or HPT (Wilk’s λ = 0.921, F [4, 193] = 4.133, *p* = 0.003, n^2^p = 0.079, β − 1 = 0.914), but not for the PPT at tibialis anterior (Wilk’s λ = 0.983, F [4, 193] = 0.834, *p* = 0.505, n^2^p = 0.017, 1 − β = 0.263). Women with chronic migraine with the Met/Met genotype exhibited reduced CPM indexes for PPT, CPT, or HPT at the temporalis (trigeminal area) than those with the Val/Val genotype. This study showed that CPM deficit is higher in women with migraine with the Met/Met genotype, but this association is mostly present in the symptomatic (trigeminal) area in the chronic form of the disease. No association of the Met/Met genotype with CPM was seen in healthy controls.

## 1. Introduction

Headaches are commonly seen in clinical practice by medical doctors. A recent study has estimated that up to 65% of the worldwide adult population will experience headaches during the coming year [1]. Migraine is one of the most prevalent primary headaches. In fact, the last Global Burden of Disease Study revealed that migraine represents a major global health challenge, showing a global pooled prevalence of 34.6% [2]. Migraine is the leading cause of disability in women younger than 50 years [3], and it is associated with social, occupational, economic and health impacts on society [4].

Several hypotheses, e.g., nociceptive, vascular or cortical, are proposed to explain the pathogenesis of migraine [5]. The nociceptive theory proposes that migraine is characterized by a sensitized state of the central nervous system [6]. A state of altered pain processing can be featured by exaggerated response (e.g., hyperalgesia/allodynia) to different stimuli (facilitatory state) and/or impaired conditioned pain modulation (CPM), i.e., inhibition of descending pain pathways (inhibitory state) [7]. Current evidence is mostly consistent in the presence of exaggerated responses to different stimuli; however, the results on deficiency in CPM in migraine is conflicting [8,9]. Both reviews concluded that conflicting results on CPM studies in migraine could be related to large variability in the CPM paradigms used, small sample sizes including both sexes and lack of differentiation between episodic and chronic migraine [8,9]. In addition, most published studies investigating CPM activation in people with migraine have not analyzed the influence of genetics (e.g., single nucleotide polymorphisms).

In fact, more than 100 genes have been potentially involved in altered pain processing [10]. Among the single-nucleotide polymorphisms (SNPs) alterations that have been proposed to be related to altered pain processing, the catechol-O-methyltransferase (COMT) rs4680 is probably the most investigated SNP in chronic pain conditions [11], including migraine [12]. The COMT is an enzyme involved in metabolic degradation of several neurotransmitters, such as dopamine, norepinephrine or epinephrine [13]. Genetic alteration of this SNP leads to a valine (Val)-to-methionine (Met) substitution at codon 158 of this gene, resulting in differences in gene activity [11]. This alteration in the Val158Met rs4680 polymorphism causes the production of a genotype (Met/Met) associated with decreased enzyme activity and high pain sensitivity [14].

Evidence about the role of Val158Met polymorphism in migraine is heterogeneous. Chen et al. found an association between COMT rs4680 polymorphism with a decreased risk of migraine [15]; whereas Liao et al. did not find an association between the Val158Met polymorphism and a higher risk of suffering from migraine [16]. The lack of an association between Val158Met polymorphism and higher risk of suffering from migraine does not exclude a potential influence of this SNP in the clinical phenotype. In fact, preliminary evidence suggests that patients with migraine carrying the Met allele experience worse migraine-associated symptoms [17] and higher-pressure pain sensitivity [18] than those patients carrying the Val allele. Vetterlein et al. concluded the presence of high heterogeneity in the association between the Met allele and altered pain processing and reported that the impact of the Val158 Met rs4680 polymorphism on pain sensitivity seems to be present when descending pain pathways are impaired [19]. No previous study has investigated the influence of Val158 Met rs4680 polymorphism on CPM in subjects with migraine [8,9]. Accordingly, to improve current knowledge on a potential effect of Val158 Met rs4680 polymorphism on pain modulation, the objective of the current study was to investigate the influence of the Val158Met rs4680 polymorphism in CPM in women with migraine. We hypothesized that women with migraine, but not healthy women, carrying the Met allele will exhibit more impaired CPM than those carrying the Val allele.

## 2. Results

### 2.1. Descriptive Data Distribution of Val158Met Polymorphism in Migraine

The sociodemographic, clinical features and Val158Met genotype distribution of the sample are shown in Table 1. Chi-square tests revealed significant differences in educational level (*p* = 0.006) and employment status (*p* < 0.001) among groups. A one-way ANOVA revealed significant intergroup differences in anxiety (*p* = 0.001) and depressive (*p* = 0.005) levels. The genotype distributions in women with and without migraine did not deviate from those expected based on the Hardy–Weinberg equilibrium. The distribution of Val158Met genotypes (*p* = 0.097) was not significantly different among women with episodic/chronic migraine and controls.

### 2.2. Effect of Val158Met Polymorphism on Mechanical CPM in the Temporalis Muscle

The repeated-measures analyses (the estimated marginal means and standard deviations can be seen in Table 2) showed a significant time effect for PPTs (Wilk’s λ = 0.930, F [1, 193] = 14.455, *p* < 0.001, n^2^p = 0.070, 1 − β = 0.966). PPT values were higher after the conditioned stimulus (mean difference: 17.5 kPa, 95% CI 12.5 to 22.6, *p* < 0.001) than before the stimulus. A significant group*time*Val158Met interaction effect (Wilk’s λ = 0.917, F [4, 193] = 4.377, *p* = 0.002, n^2^p = 0.083, 1 − β = 0.930) was found after controlling for educational level (Wilk’s λ = 0.953, F [1, 193] = 0.449, *p* = 0.718, n^2^p = 0.004, 1 − β = 0.096), employment status (Wilk’s λ = 0.999, F [1, 193] = 0.082, *p* = 0.775, n^2^p = 0.000, 1 − β = 0.059), anxiety levels (Wilk’s λ = 0.958, F [1, 193] = 1.049, *p* = 0.312, n^2^p = 0.005, 1 − β = 0.126), depressive levels (Wilk’s λ = 0.987, F [1, 193] = 1.654, *p* = 0.201, n^2^p = 0.013, 1 − β = 0.248) or prophylactic medication (Wilk’s λ = 0.999, F [1, 193] = 0.150, *p* = 0.699, n^2^p = 0.001, 1 − β = 0.067).

Before the application of the conditioned stimulus, post-hoc analyses revealed that: (1) women with chronic migraine with the Met/Met genotype had lower PPT values than those with the Val/Val (mean difference: −120.4 kPa, 95%CI −174.9 to −65.9, *p* < 0.001) and Val/Met (mean difference: −97 kPa, 95%CI −142.4 to −51.5, *p* < 0.001) genotypes; (2) women with episodic migraine with the Met/Met genotype had lower PPTs (mean difference: −61.4 kPa, 95%CI −112.6 to −10.2, *p* = 0.013) than those with Val/Val genotype; (3) healthy women with the Met/Met genotype had lower PPT values than those with the Val/Val (mean difference: −101.2 kPa, 95%CI −167.7 to −34.7, *p* = 0.001) and Val/Met (mean difference: −85.7 kPa, 95%CI −147.4 to −24.0, *p* = 0.003) genotypes.

Post-hoc analyses for data after application of the conditioned stimulus indicated that women with migraine, either episodic or chronic, carrying the Met/Met genotype had lower PPTs than women with migraine carrying the Val/Val genotype (chronic migraine mean difference: −113.7 kPa, 95% CI: −173.5 to −53.9, *p* = 0.001; episodic migraine mean difference: −73.4 kPa, 95% CI: −131.8 to −15.0, *p* = 0.008, Table 2).

### 2.3. Effect of Val158Met Polymorphism on Mechanical CPM in the Epicondyle Muscle

The MANCOVA (the estimated marginal means and standard deviations can be seen in Table 3) revealed a significant time effect for PPTs (Wilk’s λ = 0.980, F [1, 193] = 3.931, *p* = 0.049, n^2^p = 0.020, 1 − β = 0.505). PPT values were higher after the conditioned stimulus (mean difference: 14.9 kPa, 95% CI 1.5 to 11.4, *p* = 0.014) than before the stimulus. Thus, a significant group*time*Val158Met interaction effect was also observed (Wilk’s λ = 0.892, F [4, 193] = 5.870, *p* < 0.001, n^2^p = 0.108, 1 − β = 0.982) after controlling for educational level (Wilk’s λ = 0.975, F [1, 193] = 3.900, *p* = 0.068, n^2^p = 0.025, 1 − β = 0.596), employment status (Wilk’s λ = 0.994, F [1, 193] = 1.261, *p* = 0.263, n^2^p = 0.006, 1 − β = 0.201), anxiety levels (Wilk’s λ = 0.928, F [1, 193] = 3.049, *p* = 0.074, n^2^p = 0.019, 1 − β = 0.526), depressive levels (Wilk’s λ = 0.939, F [1, 193] = 1.811, *p* = 0.275, n^2^p = 0.017, 1 − β = 0.346) or prophylactic medication (Wilk’s λ = 0.992, F [1, 193] = 1.073, *p* = 0.302, n^2^p = 0.008, 1 − β = 0.177).

Before the application of the conditioned stimulus, post-hoc analyses showed that women with chronic migraine with the Met/Met genotype had lower PPT values than those with the Val/Val (mean difference: −143.0 kPa, 95%CI −209.4 to −76.6, *p* < 0.001) and Val/Met genotype (mean difference: −106.6 kPa, 95%CI −161.9 to −51.2, *p* < 0.001). After application of the conditioned stimulus, women with episodic/chronic migraine carrying the Met/Met genotype had lower PPTs than women with migraine carrying the Val/Val genotype (chronic migraine mean difference: −115.3 kPa, 95% CI: −173.1 to −57.5, *p* = 0.010; episodic migraine mean difference: −87.8 kPa, 95% CI: −160.8 to −14.8, *p* = 0.012, Table 3).

### 2.4. Effect of Val158Met Polymorphism on Mechanical CPM in the Tibialis Anterior

The analyses (the estimated marginal means, and standard deviations can be seen in Table 4) showed a significant time effect for PPTs (Wilk’s λ = 0.946, F [1, 193] = 11.036, *p* = 0.001, n^2^p = 0.054, 1 − β = 0.911). PPT values were higher after the conditioned stimulus (mean difference: 22.6 kPa, 95% CI 14.3 to 30.9, *p* < 0.001) than before the stimulus. No significant group*time*Val158Met interaction (Wilk’s λ = 0.983, F [4, 193] = 0.834, *p* = 0.505, n^2^p = 0.017, 1 − β = 0.263) was seen after controlling for educational level (Wilk’s λ = 0.975, F [1, 193] = 1.925, *p* = 0.146, n^2^p = 0.012, 1 − β = 0.394), employment status (Wilk’s λ = 0.990, F [1, 193] = 1.952, *p* = 0.164, n^2^p = 0.010, 1 − β = 0.285), anxiety levels (Wilk’s λ = 0.980, F [1, 193] = 2.665, *p* = 0.105, n^2^p = 0.020, 1 − β = 0.367), depressive levels (Wilk’s λ = 0.980, F [1, 193] = 2.644, *p* = 0.106, n^2^p = 0.020, 1 − β = 0.365) or prophylactic medication (Wilk’s λ = 0.999, F [1, 193] = 0.046, *p* = 0.830, n^2^p = 0.000, 1 − β = 0.055).

### 2.5. Effect of Val158Met Polymorphism on Thermal CPM (Cold Pain Thresholds)

The repeated-measures analyses revealed a significant group*time*Val158Met interaction effect (Wilk’s λ = 0.872, F [4, 193] = 7.111, *p* < 0.001, n^2^p = 0.128, 1 − β = 0.995, Table 5), but not a time effect (Wilk’s λ = 0.984, F [1, 193] = 3.196, *p* = 0.075, n^2^p = 0.016, 1 − β = 0.428) for CPT after controlling for educational level (Wilk’s λ = 0.994, F [1, 193] = 1.163, *p* = 0.282, n^2^p = 0.006, 1 − β = 0.189), employment status (Wilk’s λ = 0.931, F [1, 193] = 1.082, *p* = 0.144, n^2^p = 0.006, 1 − β = 0.157), anxiety levels (Wilk’s λ = 0.999, F [1, 193] = 0.004, *p* = 0.949, n^2^p = 0.000, 1 − β = 0.050), depressive levels (Wilk’s λ = 0.982, F [1, 193] = 2.355, *p* = 0.127, n^2^p = 0.018, 1 − β = 0.331) or prophylactic medication (Wilk’s λ = 0.999, F [1, 193] = 0.055, *p* = 0.815, n^2^p = 0.000, 1 − β = 0.056).

Before the application of the conditioned stimulus, post-hoc analyses showed that women with chronic migraine with the Val/Met genotype had lower CPTs than those with the Val/Val (mean difference: −4.9 °C, 95%CI −8.5 to −1.2, *p* = 0.004) and Met/Met genotype (mean difference: −7.2 °C, 95%CI −10.3 to −4.1, *p* < 0.001). Thus, after application of the conditioned stimulus, women with chronic migraine carrying the Met/Met genotype had higher CPTs values than those with the Val/Val (mean difference: 5.5 °C, 95% CI: 1.7 to 9.4, *p* = 0.002) and Val/Met genotype (mean difference: 6.7 °C, 95% CI: 3.5 to 9.9, *p* < 0.001, Table 5).

### 2.6. Effect of Val158Met Polymorphism on Thermal CPM (Heat Pain Thresholds)

The repeated-measures analyses revealed a significant group*time* Val158Met interaction effect (Wilk’s λ = 0.921, F [4, 193] = 4.133, *p* = 0.003, n^2^p = 0.079, 1 − β = 0.914, Table 6), but not a time effect (Wilk’s λ = 0.985, F [1, 193] = 2.895, *p* = 0.090, n^2^p = 0.015, 1 − β = 0.395) for HPT after controlling for educational level (Wilk’s λ = 0.994, F [1, 193] = 3.049, *p* = 0.124, n^2^p = 0.009, 1 − β = 0.356), employment status (Wilk’s λ = 0.998, F [1, 193] = 0.294, *p* = 0.588, n^2^p = 0.002, 1 − β = 0.084), anxiety levels (Wilk’s λ = 0.985, F [1, 193] = 1.913, *p* = 0.169, n^2^p = 0.015, 1 − β = 0.279), depressive levels (Wilk’s λ = 0.995, F [1, 193] = 0.601, *p* = 0.440, n^2^p = 0.005, 1 − β = 0.120) and prophylactic medication (Wilk’s λ = 0.998, F [1, 193] = 0.246, *p* = 0.620, n2p = 0.02, 1 − β = 0.078).

Before the application of the conditioned stimulus, post-hoc analyses showed that women with chronic migraine carrying the Met/Met genotype had lower HPT values than those with the Val/Met genotype (mean difference: −3.0 °C, 95%CI −5.5 to −0.5, *p* = 0.010). Furthermore, after application of the conditioned stimulus, it was found that the group of patients with chronic migraine with the Met/Met genotype had lower HPTs values than those with the Val/Val (mean difference: −2.6 °C, 95% CI: −5.3 to −0.3, *p* = 0.046) and Val/Met genotype (mean difference: −2.7 °C, 95% CI: −4.9 to −0.4, *p* = 0.011).

### 2.7. Effect of Val158Met Polymorphism on CPM Indexes

The MANCOVA analyses showed a significant group (Wilk’s λ = 0.518, F [10, 378] = 14.701, *p* < 0.001, n^2^p = 0.280, 1 − β = 0.999), and Val158Met (Wilk’s λ = 0.810, F [10, 378] = 4.196, *p* < 0.001, n^2^p = 0.100, 1 − β = 0.999) effect and a significant group*Val158Met interaction (Wilk’s λ = 0.737, F [20, 627] = 3.025, *p* < 0.001, n^2^p = 0.073, 1 − β = 0.998) after controlling for educational level (Wilk’s λ = 0.869, F [5, 189] = 1.692, *p* = 0.524, n^2^p = 0.004, 1 − β = 0.121), employment status (Wilk’s λ = 0.869, F [5, 189] = 1.708, *p* = 0.508, n^2^p = 0.005, 1 − β = 0.184), anxiety levels (Wilk’s λ = 0.970, F [5, 189] = 2.174, *p* = 0.084, n^2^p = 0.034, 1 − β = 0.621), depressive levels (Wilk’s λ = 0.975, F [5, 189] = 1.712, *p* = 0.136, n^2^p = 0.046, 1 − β = 0.431) or prophylactic medication (Wilk’s λ = 0.980, F [5, 189] = 0.523, *p* = 0.759, n^2^p = 0.020, 1 − β = 0.189).

For mechanical CPM index in the temporalis muscle (Table 7), post-hoc analyses found that (1) women with chronic migraine with the Val/Val genotype had higher CPM index values than those with the Met/Met (mean difference: 10.5%, 95%CI 0.9 to 20.0, *p* = 0.026) and Val/Met genotype (mean difference: 16.8%, 95%CI 8.8 to 24.8, *p* < 0.001); (2) pain-free women with the Met/Met genotype had higher CPM index values than those with the Val/Val (mean difference: 14.9%, 95%CI 3.3 to 26.6, *p* = 0.007) and Val/Met genotype (mean difference: 18.9%, 95%CI 8.1 to 29.8, *p* < 0.001). For mechanical CPM index in the lateral epicondyle intergroup comparisons revealed that women with episodic migraine with the Met/Met genotype had lower CPM index values than women with the Val/Val genotype (mean difference: −9.8%, 95%CI −19.8 to −0.2, *p* = 0.012). No significant intergroup differences for mechanical CPM index in the tibialis anterior muscle were observed (Table 7).

Post-hoc data obtained for the CPM index for CPT indicated that (1) women with chronic migraine carrying the Val/Val genotype had higher index (CPM activation) than those carrying the Val/Met (mean difference: 14.9%, 95%CI 1.9 to 27.9, *p* = 0.018) or Met/Met (mean difference: 14.0%, 95%CI 0.7 to 27.3, *p* = 0.035) genotype (Table 8); (2) controls with the Val/Val genotype also had higher index (CPM activation) than those with the Val/Met (mean difference: 11.3%, 95%CI 0.01 to 22.6, *p* = 0.048) and Met/Met (mean difference: 16.6%, 95%CI 0.3 to 32.8, *p* = 0.043) genotypes (Table 8). Post-hoc analysis for CPM index for HPT revealed that (1) women with chronic migraine with the Val/Val genotype had higher CPM index values than those with the Val/Met genotype (mean difference: 3.7%, 95%CI 0.5 to 6.9, *p* = 0.014); (2) women with episodic migraine with the Val/Met genotype had higher CPM index values than those with the Met/Met genotype (mean difference: 3.8%, 95%CI 1.0 to 6.7, *p* = 0.003).

## 3. Discussion

This study increases current knowledge of Val158Met rs4680polymorphism by revealing that women with episodic and chronic migraine exhibited similar CPM deficits compared with pain-free women. Thus, CPM deficits within the trigeminal area, but not distant pain-free areas, were higher in women with chronic migraine carrying the Met/Met genotype, but not in women with episodic migraine. No association of the Met/Met genotype with CPM indexes (activation) was identified in healthy controls.

Former literature on CPM in migraine is heterogeneous, but the overall conclusion is no differences in CPM between individuals with migraine and controls [8,9]. This lack of differences could be attributed to small samples, lack of characterization of migraine groups, and use of different CPM paradigms. In fact, most previous studies on CPM did not differentiate between migraine subtypes, which can be crucial for pain profile in this population [9]. The current study observed similar CPM deficits in women with episodic or chronic pain when compared to pain-free women, since no changes or even negative values of CPM activation index were identified. These results would support similar impairment in descending pain processing between chronic and episodic migraine. Thus, current data agree with a recent meta-analysis showing no significant differences in most quantitative sensory tests between episodic and chronic migraine regardless of the method used [20].

The current study focused on the influence of Val158Met rs4680 polymorphism in CPM activity. First, it should be noted that we did not observe significant differences in the distribution of Val158Met genotypes between women with episodic/chronic migraine and healthy women, in agreement with the meta-analysis by Liao et al. [16]. Our main results found that the CPM index to mechanical, but not thermal, stimuli in trigeminal, but not distant pain-free, areas was associated with the Met/Met genotype in women with chronic, but not episodic, migraine. The current findings agree with a previous study showing higher pain sensitivity to pressure pain in women with chronic, but not episodic, migraine carrying a Met/Met genotype [18] and would suggest that the Val158Met polymorphism could play a role within pain processing in the chronic form of the disease, particularly in mechanical pain sensitivity at the symptomatic area. In fact, the trigeminal (symptomatic area) was the only site showing consistent differences in pressure pain sensitivity between patients with chronic migraine and healthy controls (MD −67.36 kPa, 95%CI −101.31 to −33.42) in a previous meta-analysis [21]. Additionally, we consistently saw lower PPTs, i.e., higher pressure pain hyperalgesia, in the trigeminal area in women with chronic/episodic migraine, as well as in healthy controls, carrying the Met/Met genotype compared with those carrying the Val/Val genotype (see Table 2). Finally, higher pressure pain hyperalgesia in distant pain-free areas associated with the Met/Met genotype was just observed in women with chronic migraine, but neither in women with episodic migraine nor healthy controls. Again, widespread pressure pain hyperalgesia in women with chronic, but not episodic, migraine carrying the Met/Met genotype has been previously observed [18].

Previous studies conducted in healthy individuals [22] or in people with acute low back pain [23] did not observe an association between COMT rs4680 Val158Met genotype and CPM [22,23]. Since the impact of the Val158Met polymorphism on pain sensitivity seems to be present when descending pain pathways are impaired [19], it is possible that the inclusion of healthy people [22] or individuals with acute pain [23] would explain the lack of association due to the lack of CPM impairment in these populations. In agreement with this hypothesis, we did not observe an association between any CPM index and Val158Met genotype in our sample of healthy controls, in agreement with Korczeniewska et al. [22], Lie et al. [23] and Vetterlein et al. [19]. The lack of association between rs4680 Val158Met genotype and CPM indexes in healthy women could be explained by the fact that healthy people carrying the Met/Met genotype exhibited higher activation in cortical areas, e.g., periaqueductal gray matter, associated with descending pain inhibition when compared with healthy people carrying the Val/Val genotype [24]. In line with a higher cortical activation of areas associated with descending pain inhibition, we found a higher mechanical CPM index in the trigeminal area in healthy controls carrying the Met/Met genotype (see Table 7).

Several mechanisms can explain an association between Val158Met polymorphism and deficits in descending pain processing. For instance, the reduced COMT gene activity associated with the Met allele leads to a reduction in the content of enkephalins in cortical areas associated with pain processing [25]. In fact, migraine has been associated with a non-physiological and unbalanced production of neurotransmitters and neuromodulators, e.g., enkephalins, which promote sensitization of trigeminal neurons [26]. A second mechanism may be related to stimulation of β2-adrenergic receptors in the central nervous system due to increased catecholamine levels, associated with a reduced COMT gene activity [27]. A third potential mechanism may be that a chronic pain experience would enhance genetic sensitivity to painful stimuli, possibly through a process of epigenetic modification [28]. All these mechanisms are complexly interconnected in migraine pathogenesis.

Finally, it is suggested that COMT rs4680 polymorphism may have different effects on different pain modalities [29]. It seems that the Val158Met genotype is mainly associated with mechanical, but not thermal or electrical, stimulation, at least in healthy individuals, since data on chronic pain populations is conflicting [19]. In fact, we found higher thermal CPM indexes in women with chronic migraine carrying the Val allele compared to those carrying the Met allele (see Table 8). However, it should be noted that thermal thresholds were assessed just on the trigeminal area; hence, this association could be also explained by anatomical/symptomatic area rather than by the painful stimuli used. Future studies investigating the association of the Val158Met genotype and CPM according to different stimuli are clearly needed [19].

Although this is the first study investigating the association between Val158Met polymorphism and CPM in women with migraine, some limitations should be considered. First, the cross-sectional design did not permit us to determine a cause-and-effect direction of the current findings. Accordingly, this study should be considered as primarily associative, since it does not provide a mechanistic insight into how COMT-related catecholaminergic modulation affects descending pain inhibitory pathways. Second, the included sample just included women with/without migraine derived from a specialized tertiary hospital center. Given the increased risk of migraine in women and the biological sex-related differences in pain processing, it would be expected that women might have a different CPM response than men. Therefore, the current results should not be directly extrapolated to men. Third, medication intake was not modified, which may introduce bias in pain processing outcomes. Fourth, we only investigated the rs4680 nucleotide of Val158Met polymorphism. Future studies should include a greater number of nucleotides and other genes to further clarify their potential role in descending pain modulation in migraine.

## 4. Methods

### 4.1. Participants

An observational cross-sectional case-control study including women with/without migraine was performed. The study design followed the Strengthening Reporting of Observational studies in Epidemiology (STROBE) guidelines [30]. This study was approved by Local Ethics Committees of institutions involved (URJC_ 010220240912024; HUFA 24_117). All participants were informed and signed the informed consent before their inclusion in this study.

Consecutive women with headache attending the Neurology Department of Hospital Universitario Fundación Alcorcón (HUFA), an urban local hospital (Madrid, Spain), from March to September 2025 were potentially eligible to participate. Women with a migraine diagnosis according to the third edition (ICHD-III) of International Headache Society (IHS) [31] were included. Migraine history, including location, quality features, time with migraine and medication intake, was obtained by an experienced neurologist. Participants showing any of the following criteria were excluded: 1. presence of other primary and/or secondary headache [31]; 2. previous cervical trauma; 3. comorbid medical diseases (e.g., fibromyalgia, rheumatic conditions) affecting pain modulation; 4. psychiatric diagnoses; 5. active medication intake (e.g., antipsychotics, anticonvulsants, or anticholinergics) affecting cognition; 6. pregnancy; or, 7. having received any medical intervention, including aesthetic blocks, in the past 3 months.

A headache diary for 4 weeks was used for registering the frequency of migraine attacks (days/month), the duration of each migraine attack (hours) and the intensity of each attack (numerical pain rate scale [NPRS], 0–10 points) [32]. Thus, the Spanish version of the Hospital Anxiety and Depression Scale (HADS) was used to assess the presence of anxiety and depressive symptoms [23]. The HADS consists of 7 items evaluating anxiety symptoms (HADS-A, 0–21 points) and 7 items evaluating depressive symptoms (HADS-D, 0–21 points) [33]. The HADS has exhibited good internal consistency and reliability to be used in headache patients [34].

A control (non-migraine) group of women without a history of headache diagnosis and who had not reported a headache attack the previous year, matched by age to the migraine group, was recruited from local announcements. The inclusion in the control group was based on a clinical medical examination by a neurologist.

### 4.2. DNA Collection and COMT Genotyping

Non-stimulated saliva samples were collected in all participants following standardized procedures. Saliva was preferred instead of blood sampling because salivary collection is a non-invasive, stress-free and ethically suitable assessment method. Participants were asked not to eat, drink or chew gum for 1 h before saliva collection. Saliva sample was collected between 9 and 11 am into a specific collection tube (passive drooling technique). Immediately after collection, samples were centrifuged at 3000 rpm for 15 min to obtain the cell sediment and were stored at −20 °C until the main analysis. Technicians were blinded to the subject’s condition.

Genomic DNA was hence extracted from saliva cell sediments using the “Genomic DNA extraction and purification Kit” (Real Molecular Biology, Thermo Fisher Scientific Inc., Hemel Hempstead, Hertfordshire, UK) following the manufacturer instructions. The single Val158Met (rs4680) nucleotide polymorphism was genotyped using a TaqMan^®^ Drug Metabolism Genotyping Assays on a Real Time PCR ABI Prism 7000 Sequence Detection System (Applied Byosystem, New York, NY, USA) in the Genomic Unit at the Centro de Apoyo Tecnológico Universidad Rey Juan Carlos, Madrid (Spain). The possible haplotypes were associated with different fluorescent dyes for proper identification of the three potential different genotype forms: Val/Val (H/H), Val/Met (H/L), Met/Met (L/L). These results are derived from a G → A substitution at the following sequence:

CCAGCGGATGGTGGATTTCGCTGGC [A/G] TGAAGGACAAGGTGTGCATGCCTGA

### 4.3. Conditioned Pain Modulation

In women with episodic migraine, the evaluation was conducted when they were headache-free and when at least one week had elapsed since the last headache attack to avoid migraine-related allodynia. In those women with chronic migraine, evaluation was conducted pain-free (if possible) or when the headache intensity was <3/10 points. Thus, evaluation was conducted at least one–two days since the last headache. All participants were asked to avoid taking analgesics or muscle relaxants 48 h prior to the examination.

Changes in mechanical and thermal pain thresholds were calculated to evaluate CPM activity. Pressure pain thresholds (PPTs) were assessed bilaterally at the temporalis muscle (symptomatic area) and at the lateral epicondyle and tibialis anterior muscle (pain-free areas) with an electronic algometer (Somedic AB, Farsta, Sweden). Pressure was applied approximately at a rate of 30 kPa/s. Three repetitions at each point were made with an interval of 30 s to avoid temporal summation; the mean was calculated and used for statistical analyses. Heat (HPT) and cold (CPT) pain thresholds were bilaterally calculated on the frontalis muscle using an advanced thermal stimulator (ATS System, Medoc Pathways System, Ramat Yishai, Israel), following the method of limits [35]. Three repetitions were obtained with an interval of 30 s; the mean was calculated and used for analyses.

In the current study, we used the cold-pressor test [36] as a CPM paradigm, since it has shown good to excellent reliability when used in chronic pain [37]. The cold-pressor test consists of using cold water as the conditioned stimulus. Participants immersed one hand (the hand of the side of migraine in women with unilateral symptoms and the dominant hand in women with bilateral migraine or controls) for one minute in a water container (Huber K20-cc NR, Offenburg, Germany) at a temperature of 10 °C.

The procedure was conducted as follows. All pain thresholds were first calculated in a random order. Second, participants immersed their hand into cold water for one min. Third, pain thresholds were again calculated in random order immediately after the hand was withdrawn from the cold water following the same procedure described above. An impaired CPM is operationally defined as no change or negative change in pain thresholds obtained before and after the conditioned stimulus [38]. We calculated the absolute (kPa or °C, CPM change score) and the percentage (%, CPM activation index) changes between PPT, HPT and CPT values before and after the conditioned stimulus.

### 4.4. Sample Size Calculation

The estimated sample size was calculated using the G*Power 3.1.9.7 computer program. The following statistical parameters were entered into the ‘F tests’ for the repeated-measures MANOVA statistical test on the main outcome of this study, CPM: effect size (f2): 0.25; α: 0.05; statistical power: 0.90; number of groups: 3; number of measures: 2. According to these parameters, the necessary sample size identified was 206 participants.

### 4.5. Statistical Analysis

All statistical analyses were conducted using SPSS Statistical Software 25.0. Means (standard deviations) are provided for continuous variables whereas frequencies (percentages) are provided for categorical variables. Outliers were assessed throughout boxplots according to the 1.5 × interquartile range (IQR) criterion. No outliers were identified; therefore, no observations were removed or winsorized prior to analysis. Assumptions of normality were evaluated using the Shapiro–Wilk test, together with visual inspection of Q–Q plots, which indicated that the assumption of normality was met. The assumption of sphericity for repeated-measures analyses was assessed using Mauchly’s test, which indicated that the assumption of sphericity was met.

Prior to performing the MANOVA/MANCOVA analyses, the distributional assumptions of the dependent variables were examined. Univariate normality was evaluated through skewness and kurtosis coefficients, graphical inspection of histograms and Q–Q plots, as well as the Kolmogorov–Smirnov test. These assessments indicated that the data were approximately normally distributed, with no meaningful departures from normality. Multivariate normality was assessed by examining potential multivariate outliers using Mahalanobis distance. No cases exceeded the critical χ^2^ threshold based on the number of dependent variables, suggesting the absence of influential multivariate outliers and supporting the assumption of multivariate normality. Equality of variance–covariance matrices across groups was tested using Box’s M test, which showed no statistically significant differences. Finally, the assumption of homogeneity of regression slopes was checked by testing the interaction between covariates and grouping factors within the MANCOVA framework.

Then, several repeated-measures MANCOVA were conducted to determine the effect of group (episodic migraine, chronic migraine, pain-free women), and Val158Met genotype (Val/Val, Val/Met, Met/Met) on the dependent variable (pain thresholds before/after the conditioned stimulus) was conducted. Pain thresholds (PPT, HPT, CPT) were the dependent variables, time (before/after conditioned stimulus) was the within-subjects factor, and group (episodic migraine, chronic migraine, controls) and Val158Met genotype (Val/Val, Val/Met, Met/Met) were the between-subjects factors. Additionally, a multivariate analysis of covariance (MANCOVA) was conducted with group (episodic migraine, chronic migraine, healthy control) and Val158Met genotype (Val/Val, Val/Met, Met/Met) as independent variables and CPM activation index on each point (mechanical for temporalis, lateral epicondyle and tibialis anterior; thermal for the frontalis area) as the dependent variable were conducted to assess the effect of Val158 Met polymorphism. Thus, educational level, employment status, anxiety/depressive levels and prophylactic treatment were included as covariates. For post-hoc analyses, a *p* value < 0.015 (0.05/3) was considered as statistically significant (correction for multiple comparisons). Thus, partial eta squared (n^2^p) was calculated to determine effect sizes: 0.01 as a small effect, 0.06 as a medium effect and above 0.14 as a large effect.

## 5. Conclusions

This study found an effect of rs4680 Val158Met polymorphism on CPM using a mechanical or thermal stimulus within the trigeminal area in women with chronic, but not episodic, migraine carrying the Met/Met genotype. No effect on CPM in distant pain-free areas was found. Thus, no association of the Met/Met genotype with CPM activation was observed in our sample of pain-free women.

## Figures and Tables

**Table 1:** Clinical and sociodemographic data of the sample.

	Chronic Migraine (n = 70)	Episodic Migraine (n = 70)	Controls (n = 70)		
	Mean (SD)	Mean (SD)	Mean (SD)	F	*p*
Age (years)	46.1 (5.2)	48.1 (7.2)	49.1 (9.6)	2.905	0.057
Intensity (NPRS, 0–10)	8.6 (1.4)	7.6 (1.55)	---		
Frequency (days/month)	16.5 (6.4)	3.5 (2.7)	---		
Duration (hours/attack)	33.1 (20.3)	25.3 (23.9)	---		
Anxiety (HADS-A, 0–21)	8.05 (4.4)	9.3 (3.8)	6.8 (3.1)	7.592	0.001
Depression (HADS-D, 0–21)	5.05 (3.6)	5.6 (3.7)	3.7 (3.2)	5.453	0.005
	n (%)	n (%)	n (%)	χ^2^	*p*
Side of migraine					
Left	12 (17%)	14 (20%)	---	1.602	0.445
Right	19 (27%)	17 (24%)	---		
Bilateral	39 (56%)	39 (56%)	---		
Preventive treatment					
Yes (amitriptyline)	52 (74.3%)	48 (68.6%)	---		
No	18 (25.7%)	22 (31.4%)	---	0.560	0.454
Educational level				14.577	0.006
Primary	0 (0%)	4 (5.7%)	4 (5.7%)		
Secondary	31 (44.3%)	16 (22.9%)	14 (20%)		
Higher education	39 (55.7%)	50 (71.4%)	52 (74.30%)		
Employment status					
Student	6 (8.6%)	3 (4.3%)	0 (0%)	24.400	<0.001
Working	52 (74.3%)	56 (80%)	54 (77.1%)		
Unemployed	12 (17.1%)	7 (10%)	4 (5.7%)		
Retired	0 (0%)	4 (5.7%)	12 (17.1%)		
Marital status					
Single	25 (35.7%)	26 (37.1%)	20 (28.6%)		
Married	33 (47.1%)	36 (51.4%)	36 (51.4%)	4.545	0.603
Divorced	6 (8.6%)	2 (2.9%)	8 (11.4%)		
Widowed	6 (8.6%)	6 (8.6%)	6 (8.6%)		
Val158Met genotype				7.856	0.097
Val/Val	15 (21.43%)	21 (30.00%)	21 (31.25%)		
Val/Met	31 (44.28%)	28 (40.00%)	38 (54.69%)		
Met/Met	24 (34.29%)	21 (30.00%)	11 (14.06%)		

n: number of subjects, SD: standard deviation; NPRS: Numerical Pain Rating Scale; HADS: Hospital Anxiety and Depression Scale (A: anxiety, D: depression).

**Table 2:** Effect of Val158Met polymorphism on conditioned pain modulation in the temporalis muscle.

	Chronic Migraine		Episodic Migraine		Pain-Free Controls	
	Before Stimulus	After Stimulus	Before Stimulus	After Stimulus	Before Stimulus	After Stimulus
	Mean (SD)	Mean (SD)	Mean (SD)	Mean (SD)	Mean (SD)	Mean (SD)
Val/Val	343.6 (13.9)	342.5 (15.9)	308.4 (15.0)	312.0 (17.1)	390.1 (15.3)	450.3 (17.5)
Val/Met	319.6 (12.5)	288.8 (14.2)	282.5 (12.9)	274.7 (14.7)	374.6 (11.5)	421.4 (13.1)
Met/Met	223.2 (17.7)	228.8 (20.3)	246.9 (14.8)	238.6 (16.9)	288.9 (22.7)	377.6 (26.0)
F	19.070	5.886	4.295	4.610	7.203	2.728
*p*	<0.001	0.003	0.014	0.011	0.001	0.068
n^2^p	0.165	0.057	0.044	0.046	0.069	0.027
1 − β	0.999	0.871	0.749	0.774	0.931	0.534

**Table 3:** Effect of Val158Met polymorphism on conditioned pain modulation in the lateral epicondyle.

	Chronic Migraine		Episodic Migraine		Pain-Free Controls	
	Before Stimulus	After Stimulus	Before Stimulus	After Stimulus	Before Stimulus	After Stimulus
	Mean (SD)	Mean (SD)	Mean (SD)	Mean (SD)	Mean (SD)	Mean (SD)
Val/Val	389.0 (21.6)	349.2 (25.3)	370.2 (18.3)	368.6 (21.4)	444.1 (27.7)	504.0 (32.5)
Val/Met	352.6 (15.2)	312.4 (17.8)	335.8 (15.7)	332.1 (18.4)	391.7 (14.0)	443.2 (16.4)
Met/Met	246.0 (17.0)	233.9 (19.9)	311.7 (18.1)	280.7 (21.2)	399.8 (18.7)	441.4 (21.9)
F	16.952	4.843	2.592	4.295	1.424	1.534
*p*	<0.001	0.009	0.077	0.014	0.243	0.218
n^2^p	0.149	0.048	0.026	0.044	0.015	0.016
1 − β	0.999	0.796	0.512	0.748	0.303	0.324

**Table 4:** Effect of Val158Met polymorphism on conditioned pain modulation in the tibialis anterior muscle.

	Chronic Migraine		Episodic Migraine		Pain-Free Controls	
	Before Stimulus	After Stimulus	Before Stimulus	After Stimulus	Before Stimulus	After Stimulus
	Mean (SD)	Mean (SD)	Mean (SD)	Mean (SD)	Mean (SD)	Mean (SD)
Val/Val	404.2 (25.3)	402.6 (31.5)	401.9 (21.4)	404.0 (26.7)	508.4 (21.8)	567.5 (27.2)
Val/Met	397.3 (17.8)	389.3 (22.2)	356.0 (18.4)	356.4 (23.0)	534.2 (16.4)	597.3 (20.4)
Met/Met	292.7 (19.9)	300.8 (24.8)	335.3 (21.2)	324.9 (26.4)	530.3 (32.4)	621.6 (40.4)
F	9.481	4.664	2.560	2.236	0.456	0.699
*p*	<0.001	0.011	0.080	0.110	0.635	0.498
n^2^p	0.089	0.046	0.026	0.023	0.005	0.007
1 − β	0.979	0.780	0.507	0.452	0.124	0.167

**Table 5:** Effect of Val158Met polymorphism on conditioned pain modulation with cold pain thresholds.

	Chronic Migraine		Episodic Migraine		Pain-Free Controls	
	Before Stimulus	After Stimulus	Before Stimulus	After Stimulus	Before Stimulus	After Stimulus
	Mean (SD)	Mean (SD)	Mean (SD)	Mean (SD)	Mean (SD)	Mean (SD)
Val/Val	16.9 (1.2)	13.6 (1.2)	16.9 (1.0)	17.3 (1.0)	14.9 (1.5)	13.3 (1.6)
Val/Met	11.9 (0.8)	12.4 (0.8)	18.6 (0.8)	17.8 (0.9)	14.0 (0.7)	13.4 (0.8)
Met/Met	19.1 (0.9)	19.1 (0.9)	17.5 (1.0)	18.6 (1.0)	14.0 (1.0)	15.0 (1.0)
F	16.376	13.961	0.859	0.391	0.134	0.830
*p*	<0.001	<0.001	0.425	0.677	0.875	0.438
n^2^p	0.145	0.126	0.009	0.004	0.001	0.009
1 − β	0.999	0.998	0.196	0.112	0.070	0.191

**Table 6:** Effect of Val158Met polymorphism on conditioned pain modulation with heat thresholds.

	Chronic Migraine		Episodic Migraine		Pain-Free Controls	
	Before Stimulus	After Stimulus	Before Stimulus	After Stimulus	Before Stimulus	After Stimulus
	Mean (SD)	Mean (SD)	Mean (SD)	Mean (SD)	Mean (SD)	Mean (SD)
Val/Val	41.7 (0.9)	43.3 (0.8)	42.8 (0.8)	42.9 (0.7)	42.9 (0.8)	43.8 (0.7)
Val/Met	43.3 (0.6)	43.3 (0.6)	41.7 (0.7)	42.5 (0.6)	43.0 (0.6)	44.6 (0.5)
Met/Met	40.2 (0.7)	40.6 (0.6)	43.8 (0.8)	43.0 (0.7)	43.3 (1.2)	44.0 (1.1)
F	4.465	5.149	1.900	0.154	0.045	0.442
*p*	0.013	0.007	0.152	0.858	0.956	0.643
n^2^p	0.044	0.051	0.019	0.002	0.000	0.005
1 − β	0.760	0.821	0.392	0.074	0.057	0.121

**Table 7:** Effect of Val158Met polymorphism on mechanical conditioned pain modulation indexes.

	Temporalis Muscle			Lateral Epicondyle			Tibialis Anterior Muscle		
	Chronic Migraine	Episodic Migraine	Pain-Free Controls	Chronic Migraine	Episodic Migraine	Pain-Free Controls	Chronic Migraine	Episodic Migraine	Pain-Free Controls
	Mean (SD)	Mean (SD)	Mean (SD)	Mean (SD)	Mean (SD)	Mean (SD)	Mean (SD)	Mean (SD)	Mean (SD)
Val/Val	6.3 (2.4)	0.6 (2.6)	16.7 (2.6)	−9.8 (3.4)	−0.2 (2.9)	10.8 (3.0)	0.4 (3.3)	1.3 (2.7)	11.1 (2.8)
Val/Met	−10.4 (2.1)	−2.5 (2.2)	12.7 (2.0)	−11.9 (2.4)	−1.7 (2.5)	13.5 (2.2)	−1.6 (2.3)	−0.2 (2.4)	10.9 (2.1)
Met/Met	−4.1 (3.1)	−3.0 (2.6)	31.7 (3.9)	−4.9 (2.7)	−10.0 (2.9)	12.7 (4.4)	2.4 (2.6)	−2.0 (2.7)	15.7 (4.2)
F	13.118	0.598	8.987	0.699	4.313	0.260	0.689	0.364	0.526
*p*	<0.001	0.551	<0.001	0.501	0.015	0.771	0.503	0.695	0.592
n^2^p	0.120	0.006	0.085	0.006	0.043	0.003	0.007	0.004	0.005
1 − β	0.997	0.149	0.972	0.860	0.723	0.091	0.165	0.108	0.136

**Table 8:** Effect of Val158Met polymorphism on thermal (cold and heat pain thresholds) conditioned pain modulation index.

	Cold Pain Thresholds			Heat Pain Thresholds		
	Chronic Migraine	Episodic Migraine	Controls	Chronic Migraine	Episodic Migraine	Controls
	Mean (SD)	Mean (SD)	Mean (SD)	Mean (SD)	Mean (SD)	Mean (SD)
Val/Val	−10.8 (4.3)	2.0 (3.6)	−9.9 (5.5)	3.9 (1.0)	0.1 (0.9)	2.4 (0.9)
Val/Met	4.1 (3.0)	−4.0 (3.1)	−4.7 (2.8)	0.1 (0.7)	1.9 (0.7)	4.0 (0.6)
Met/Met	3.2 (3.4)	7.1 (3.6)	6.6 (3.7)	0.9 (0.8)	−1.9 (0.8)	1.5 (1.3)
F	4.352	2.753	4.245	4.268	5.498	1.903
*p*	0.014	0.066	0.015	0.015	0.005	0.152
n^2^p	0.043	0.028	0.042	0.042	0.054	0.019
1 − β	0.749	0.538	0.737	0.739	0.847	0.392

## Data Availability

The materials and analysis code for this study are not available in any repository; however, we will make our data accessible upon request to the corresponding author.
